# Editorial June 2020

**DOI:** 10.1093/braincomms/fcaa106

**Published:** 2020-08-04

**Authors:** Tara Spires-Jones

**Affiliations:** Edinburgh, UK

## Abstract

**Graphical Abstract:**
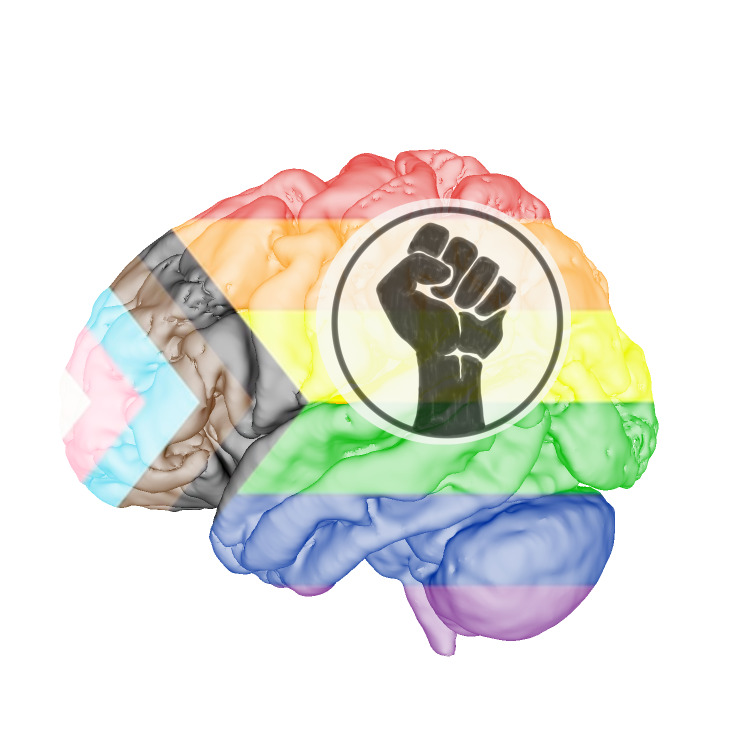

Welcome to our new issue of *Brain Communications*, Volume 2, Issue 2. We have just passed our first birthday as a journal, and what a year it has been. We have had a lot of success so far in Volume 2 and published fascinating papers. To name but a few, we have published work demonstrating that exercise exposes latent Huntington’s disease pathology (Steventon *et al.*, 2020), a study linking Alzheimer’s risk gene *BIN1* to synaptic tau accumulation (Glennon *et al.*, 2020), our first field potential article describing translational neuroscience from a PhD student perspective (Davies *et al.*, 2020), and a review on nodding syndrome, an epileptic disorder that occurs in clusters in sub-Saharan Africa (Olum *et al.*, 2020). We have also been working closely with our sister journal *Brain* and now have a shared YouTube channel for video abstracts https://www.youtube.com/channel/UClJJHH2xKQk8uZTC7-mJICg. Our cover for this new issue comes from Bevan and colleagues and shows lovely filled cells in a mouse hippocampal slice in which they observed that OPA1 protein is important for maintaining synaptic structure and function during ageing (Bevan *et al.*, 2020).

Although we have had many successes *at Brain Communications*, 2020 has been a difficult year so far, to put it mildly. The global COVID-19 pandemic has affected all areas of life including publishing, neuroscience and neurology. I am very grateful to our authors, readers, reviewers and editors who have continued to support the journal despite numerous difficulties including working in frontline clinics, educating our children in lockdown, working from home, managing lab closures, and of course illness and loss. In addition to respiratory illness, COVID-19 can also have neurological consequences from the loss of smell and taste to effects on cognitive function. Two of our Editors have written about these neurological effects of coronavirus in *Brain Communications* recently (Lleó and Alcolea, 2020; Ritchie *et al.*, 2020).

2020 has also brought into stark light the continuing systematic racism that still pervades our societies with the tragic killing of George Floyd and the health disparities in outcomes of COVID-19. This systematic racism is present in neuroscience and publishing. Our publisher, Oxford University Press (OUP), has been working with the Royal Society of Chemistry to set new standards to ensure a more inclusive and diverse culture within scholarly publishing (https://www.rsc.org/new-perspectives/talent/joint-commitment-for-action-inclusion-and-diversity-in-publishing). At *Brain Communications*, we look forward to working with OUP to help implement some of the actions highlighted in this report. Another small way that we support diversity in the field is through helping fund (via the Guarantors of *Brain*), the In2Science programme which promotes social mobility and diversity in STEM by enabling young people from disadvantaged backgrounds to interact with scientists (https://in2scienceuk.org/partners/our-supporters/). While these are useful initiatives, it is clear that we have a long way to go to make neuroscience and scientific publishing truly inclusive, and we will continue to try to listen, learn and think of ways to play our part at *Brain Communications*. While we are on the topic of inclusion, I also want to wish everyone a happy belated PRIDE month, as I am writing this in June. One of the best things about my career so far has been interacting with and learning from people of many different backgrounds, and at *Brain Communications* we strongly believe that diversity improves science, a belief backed up by evidence. I cite one nice reference here but there are many others supporting the benefits of diverse groups to scientific studies (Swartz *et al.*, 2019). In the graphical abstract included with this editorial, I've tried to summarize our support for diversity with a Pride flag and Black Lives Matter decorated brain. Although I should clearly not quit my day job, it was fun to develop a graphic to try and show support for inclusivity!

I hope that the last half of 2020 brings more rigorous science and less disaster for all of us. Thank you for reading this and supporting *Brain Communications*.

Stay safe everyone.
